# SVTR-MG: an optical character recognition network for food packaging spray codes

**DOI:** 10.1038/s41598-025-31995-y

**Published:** 2025-12-12

**Authors:** Sanbo Pan, Peng Wang

**Affiliations:** School of Electrical Engineering, Shanghai Dianji University, 300 Shuihua Road, Pudong New District, Shanghai, 201306 Shanghai China

**Keywords:** SVTR, Optical character recognition, Scene text recognition, Transformer, PaddleOCR, Engineering, Materials science, Mathematics and computing

## Abstract

Spray codes on product packaging play a critical role in food traceability, quality control, and anti-counterfeiting verification. However, accurate recognition of spray codes in industrial environments remains a significant challenge due to factors such as small character regions, fluctuating print quality, reflective packaging materials, and character deformation. To address these issues, this paper proposes a lightweight improved network named SVTR-MG. The model incorporates a Multi-scale Dilated Feature Aggregation (MDFA) module, which leverages convolutions with varying dilation rates to expand the receptive field and effectively integrate global and local features, thereby enhancing the perception of characters under multi-scale and complex background conditions. Additionally, a Global Context Self-Attention (GCSA) module is introduced, which combines channel and spatial attention mechanisms to model long-range dependencies between characters, improving the network’s robustness to uneven illumination and structural distortions. Furthermore, a dynamic dictionary mapping mechanism is proposed to optimize output alignment during the decoding phase. Experimental results demonstrate that SVTR-MG achieves a recognition accuracy of 93.2% at an inference speed of 142 FPS in complex industrial scenarios, outperforming mainstream OCR methods by approximately 5%, and meeting the real-time and accuracy requirements for deployment in production environments.

## Introduction

**Fig. 1:**
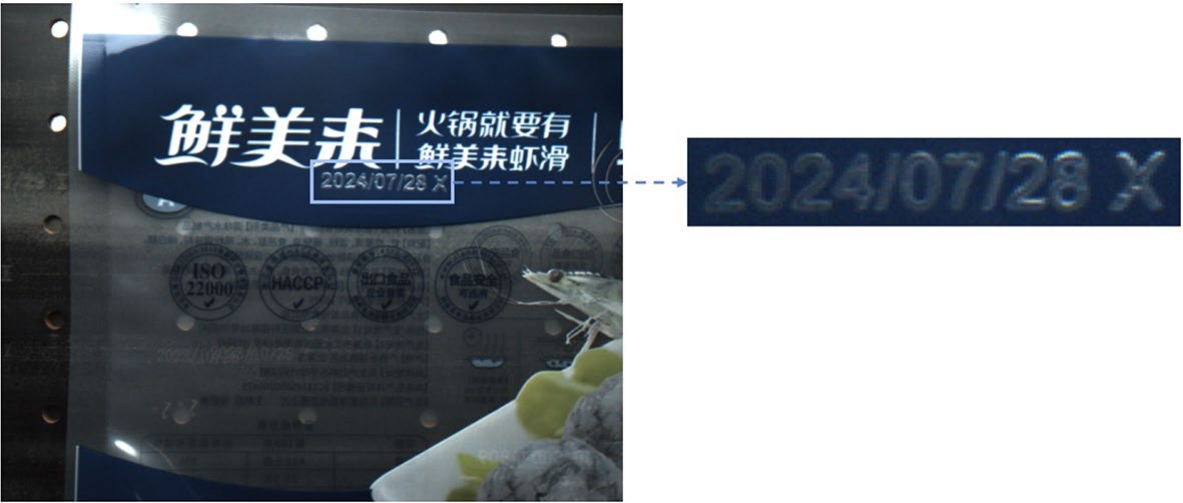
Instances of food packaging spray codes.

Food packaging codes play a crucial role in food safety, quality control, and anti-counterfeit verification^1,2^. As shown in the Fig. 1, these codes carry key information such as production dates and batch numbers, which are essential not only for quality control and traceability during manufacturing but also for ensuring consumer safety. Currently, spray printing technology remains the dominant method for marking product information within designated areas. However, accurately recognizing these codes, especially in complex industrial environments, remains a challenging problem that needs to be addressed.

Traditional spray code recognition approaches primarily rely on rule-based templates or handcrafted feature extraction^3^, which often suffer from low efficiency and poor adaptability. The presence of blurred images, uneven lighting, and character misalignment caused by high-speed production lines further exacerbates recognition difficulties. Thus, improving both recognition accuracy and processing speed is critical for the practical deployment of spray code recognition systems in the food packaging industry. The recognition of food packaging spray codes faces the following challenges:

1. Spray codes on food packaging are often printed on complex backgrounds. The market offers a wide variety of food packaging, with each brand having different packaging styles. The position of spray codes on packaging is random and may be tilted. The color of the spray codes can be similar to the background color, resulting in low contrast and making the codes blurry and hard to distinguish.
2. Differences in production processes of various food packaging, such as changes in temperature and pressure, may lead to variations in the style or shape of spray codes. Additionally, factors like oxidation or abrasion caused by improper storage may cause the codes to become smeared or partially missing.
3. Lighting conditions affect the imaging quality of spray codes. Uncontrollable factors such as imaging angle, distance, and lighting intensity can reduce image clarity and blur texture details, further increasing recognition difficulty.
4. Food packaging production lines generally operate at a fast pace, requiring the algorithm to complete text recognition on nearly 100 images within 1 second.

With the rapid advancement of deep learning and computer vision, scene text recognition^4–7^ has achieved remarkable progress. Convolutional Recurrent Neural Network (CRNN)^8^ models, which integrate Deep Convolutional Neural Networks (DCNN)^9,10^ with Recurrent Neural Networks (RNN)^11^, have enabled end-to-end feature extraction and sequence modeling without relying on traditional preprocessing steps such as binarization or character segmentation. CRNNs have demonstrated strong performance in handling blurred and complex text. However, they still struggle with long or highly distorted sequences, diverse fonts, and character adhesion.

To address these limitations, attention-based models have gained popularity in the scene text recognition community. For instance, the SEED^12^ model enhances semantic understanding by integrating a pretrained language model within an encoder–decoder framework. ViTSTR^13^ leverages a pure vision transformer^14–16^,structure to represent text images as sequential embeddings, improving both global feature extraction and inference speed. Despite their success, these models typically involve high computational costs and exhibit limited robustness when dealing with severely distorted or irregular texts.

Current scene text recognition^17^ approaches can be broadly categorized into two paradigms: sequence-based encoder–decoder frameworks^18,19^ and character detection-based segmentation methods^20,21^. In the former, representative models such as ASTER^22^ and NRTR^23^ use CNN–BiLSTM^24,25^ architectures to encode contextual dependencies and apply attention-based decoding for sequence prediction. While effective for short text, their performance degrades significantly on long text sequences-for example, recognition accuracy drops to 50% when the sequence length exceeds 14 characters-revealing the limitations of RNNs in long-range modeling^26^.

To mitigate these challenges, character segmentation-based methods^20,21^ have emerged as an alternative. For example, Char-Net^20^ applies hierarchical attention to localize and rectify each character, combined with lightweight word-level encoders and local spatial transformers for efficient recognition. Similarly, spatial transformer networks proposed by Shu et al.^27^ improve geometric robustness but at the cost of increased model complexity. Lightweight models such as those proposed by Du et al.^21^ have shown promising parameter efficiency; however, their reliance on predefined character patterns limits adaptability to complex text layouts. Notably, attention-guided CTC^28^ frameworks have recently emerged as a promising solution by combining the fast decoding capability of CTC with enhanced contextual modeling.

In recent years, Transformer^14^-based architectures have attracted significant attention in the field of scene text recognition. Their ability to model multi-scale and long-range dependencies has helped overcome many of the shortcomings of traditional OCR systems. The SVTR model^29,30^, in particular, utilizes hierarchical attention and multi-level contextual features, offering a good balance between robustness and efficiency. Nevertheless, achieving high recognition accuracy while maintaining real-time performance remains a core technical challenge for industrial applications^31^.

To tackle this challenge, we propose an enhanced SVTR-MG network tailored for spray code recognition in complex industrial scenarios. The proposed architecture incorporates two key modules: the Multi-scale Dense Feature Aggregation (MDFA) module and the Global Channel–Spatial Attention (GCSA) module. The MDFA module employs multiple atrous convolutional branches to extract multi-scale features, thereby improving the model’s sensitivity to small characters and local deformations. The GCSA module combines channel attention, channel shuffling^32^, and spatial attention mechanisms^33,34^ to capture long-range dependencies and mitigate issues caused by uneven lighting and distorted spray codes. Furthermore, a dynamic dictionary mapping mechanism is introduced to optimize the decoding process, enhancing both recognition accuracy and inference speed. Experimental results demonstrate that the proposed SVTR-MG network achieves a recognition accuracy of 93.2% on a real-world industrial dataset, outperforming existing OCR methods and meeting the real-time requirements of production line deployment. These results validate the effectiveness and practical potential of the proposed approach. The innovative contributions of this work include:

1. A novel lightweight SVTR-MG network model is proposed, which achieves a recognition accuracy of 93.2% for spray codes in complex industrial scenarios while maintaining high inference speed (142 FPS). This represents an improvement of approximately 5% over mainstream OCR methods and effectively meets the deployment requirements of real production lines.
2. The integration of a Multi-scale Dense Feature Aggregation (MDFA) module and a Global Contextual Self-Attention (GCSA) module is presented. The MDFA enhances local perception through dilated convolutions and global feature extraction, improving robustness in recognizing small characters and distorted structures. The GCSA employs a collaborative channel and spatial attention mechanism to model global feature dependencies.
3. A complex and production-relevant food packaging spray code dataset comprising 10,481 images was constructed, capturing challenging conditions such as character deformation, blur, and occlusion. Systematic evaluation on this dataset demonstrates that the proposed method achieves a recognition accuracy of 93.2%, validating its robustness and practical applicability in real-world scenarios.

The structure of this paper is as follows: section “Methodology” provides a detailed description of the design and implementation of the proposed SVTR-MG model. Section “Experiments and results” presents the experimental results and compares them with traditional methods and state-of-the-art models. Finally, section “Conclusion” summarizes the main findings of this study and discusses future research directions.

## Methodology

### SVTR network

**Fig. 2:**
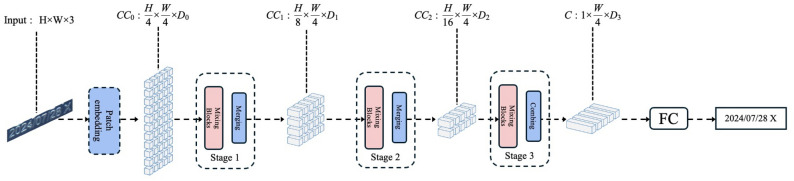
SVTR Network. This figure illustrates the overall architecture of the SVTR model, including feature extraction, attention modules, and the recognition head.

The SVTR network is built on a single-visual-model^29,30^ architecture and utilizes a multi-level component perception mechanism to achieve efficient scene text recognition. As illustrated in Fig. 2, the network follows a three-stage progressive feature processing pipeline: , the network follows a three-stage progressive feature processing pipeline.

First, the input image ($H \times W \times 3$) is decomposed into $H/4 \times W/4$ character components using a progressive overlapping patch embedding module, with each component corresponding to a local character region.

Next, a sequence of hybrid blocks is employed for multi-granularity feature extraction. The local hybrid blocks use $7 \times 11$ windowed self-attention to capture stroke-level features, while the global hybrid blocks model inter-character semantic associations through full-component interactions.

Finally, a sequence feature of size $1 \times W/4$ is produced through aggressive height compression and fusion operations, followed by parallel decoding performed via a linear classifier.

This network introduces three key innovations:

1. The progressive overlapping patch embedding replaces the conventional linear projection with two layers of $3 \times 3$ convolution, thereby enhancing the representation capability for irregular text;
2. The alternating strategy of local-global hybrid blocks prioritizes local structure extraction before establishing global associations;
3. A multi-scale feature pyramid is constructed through stepwise compression along the height dimension ($32 \rightarrow 16 \rightarrow 8 \rightarrow 1$), facilitating cross-character contextual awareness.

This design departs from traditional sequence-based models, significantly improving inference efficiency while maintaining high recognition accuracy.

### Proposed SVTR-MG network

To address the specific challenges of spray code recognition on food packaging in industrial settings, this paper introduces the SVTR-MG network (SVTR with Multi-scale and Global Context). Traditional text recognition models often struggle with false detections or omissions when confronted with complex disturbances such as variable printing quality, reflective packaging materials, and character deformation.

**Fig. 3:**
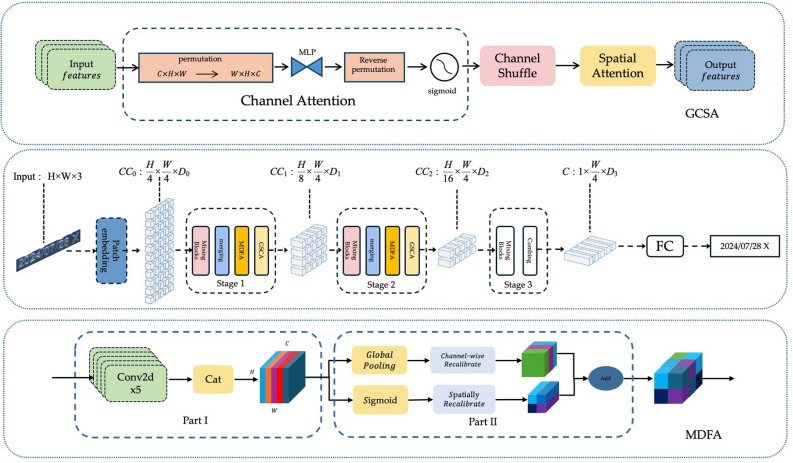
SVTR-MG Network. This figure illustrates the full architecture of the SVTR-MG model including multi-scale feature extraction layers, channel-spatial attention fusion mechanism, and the final recognition head. The long caption explains each component and its role in enhancing scene text recognition performance.

To overcome these challenges, SVTR-MG is built upon a lightweight visual Transformer architecture and incorporates two core components: a Multi-scale Dense Feature Aggregation (MDFA) module and a Global Context Self-Attention (GCSA) module. These modules enable the collaborative optimization of local details and global semantics. The overall pipeline of the proposed framework is shown in Fig. 3, which presents the multi-scale feature extraction process and the global context enhancement strategy adopted in the encoder.

The network adopts an encoder-decoder architecture. On the encoder side, stacked MDFA modules extract multi-scale local texture features from spray codes, while the GCSA module captures long-range dependencies between characters. On the decoder side, multi-level features are integrated and recognition results are generated through dynamic dictionary mapping.

This design significantly improves recognition robustness in extreme scenarios such as broken, blurred, or curved spray codes, while maintaining computational efficiency.

#### MDFA module

The Multi-Scale Dilated Fusion Attention (MDFA) module aims to enhance the multi-granularity feature representation of text regions by employing parallel feature extraction with varying dilation rates and a channel-space attention collaboration mechanism. This design captures both the microscopic structural features of character strokes (small dilation rates) and the macroscopic semantic relationships at the text-line level (large dilation rates). By integrating a dynamic attention-weight fusion strategy, it addresses the challenges of blurry local details and fragmented global context in complex text scenarios.

**Fig. 4:**
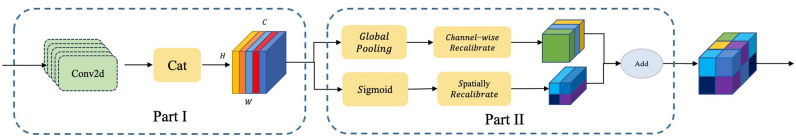
The Multi-Scale Dilated Fusion Attention (MDFA) module. This figure illustrates the architecture and working mechanism of the MDFA module, highlighting how multi-scale features are fused and recalibrated to enhance text recognition performance.

The structure of the MDFA module is illustrated in Fig. 4, which shows how multi-scale dilated convolutions and channel–spatial attention collaborate to refine both local and global feature cues.

Its ability to collaboratively model multi-scale features is crucial for text recognition tasks, effectively tackling issues such as learning deformed features in curved text, suppressing boundary interference in densely arranged characters, and restoring stroke continuity in low-resolution document images. This significantly improves robustness against unconventional layouts, blurry degradation, and background noise interference.

The MDFA module adopts a two-stage hierarchical architecture consisting of multi-scale dilated convolution for feature extraction and a collaborative fusion of channel and spatial attention mechanisms.

***Multi-scale dilated convolutional feature extraction layer:***

A five-branch heterogeneous convolutional structure is constructed to extract features across multiple receptive fields:

- **Branch 1:** A $1 \times 1$ standard convolution is used to extract local structural features while preserving the spatial resolution of the input feature map. This operation can be expressed as:
  1 $$\begin{aligned} X_1 = \text {Conv}_{1 \times 1}(X) \in \mathbb {R}^{B \times C \times H \times W} \end{aligned}$$
  where *X* is the input feature map and $X_1$ is the output after applying the $1 \times 1$ convolution.
- **Branches 2–4:** $3 \times 3$ dilated convolutional kernels are configured with increasing dilation rates of 6, 12, and 18, respectively, to capture multi-scale context. These operations are expressed as:
  2 $$\begin{aligned} X_2 = \text {Conv}_{3 \times 3, d=6}(X) \in \mathbb {R}^{B \times C \times H \times W} \end{aligned}$$
  3 $$\begin{aligned} X_3 = \text {Conv}_{3 \times 3, d=12}(X) \in \mathbb {R}^{B \times C \times H \times W} \end{aligned}$$
  4 $$\begin{aligned} X_4 = \text {Conv}_{3 \times 3, d=18}(X) \in \mathbb {R}^{B \times C \times H \times W} \end{aligned}$$
  where *d* refers to the dilation rate, and $X_2$, $X_3$, and $X_4$ represent the outputs from dilated convolutions with different dilation rates.
- **Branch 5:** A global average pooling layer aggregates global contextual information. This operation is expressed as:
  5 $$\begin{aligned} X_5 = \text {GlobalAvgPool}(X) \in \mathbb {R}^{B \times C \times 1 \times 1} \end{aligned}$$
  where $X_5$ is the output feature map after global average pooling, capturing global contextual features.

***Channel-spatial attention collaborative fusion mechanism:***

After aggregating the multi-scale features via channel-wise concatenation, a dual-path attention recalibration is performed:

- **Channel attention submodule:** A squeeze-and-excitation (SE) structure is employed to adaptively learn channel-wise weights. Specifically, global average pooling is applied to the aggregated features to obtain channel-wise statistical descriptors, which are passed through two fully connected layers (with an intermediate dimension of *C*/16, ReLU activation, and a final Sigmoid function) to generate a channel attention mask. This mask is used to recalibrate the features via channel-wise multiplication:
  6 $$\begin{aligned} A_{\text {channel}} = \sigma \left( \text {FC}_{2}\left( \text {ReLU}\left( \text {FC}_{1}(Y_{\text {avg}})\right) \right) \right) \in \mathbb {R}^{B \times C \times 1 \times 1} \end{aligned}$$
- **Spatial attention submodule:** Cross-channel feature aggregation is conducted using both max-pooling and average-pooling operations to produce a spatial saliency map. A $1 \times 1$ convolution (with Sigmoid activation) then generates the position-sensitive spatial attention map:
  7 $$\begin{aligned} A_{\text {space}} = \sigma (\text {Conv}_{1 \times 1}(Y_{\text {space}})) \in \mathbb {R}^{B \times 1 \times H \times W} \end{aligned}$$
  Element-wise multiplication enhances spatially significant regions of the feature map:
  8 $$\begin{aligned} X_{\text {out spatial}} = X \times A_{\text {space}} \in \mathbb {R}^{B \times C \times H \times W} \end{aligned}$$

***Feature fusion and optimization:***

The outputs from the channel and spatial attention branches are fused through element-wise addition:

9 $$\begin{aligned} X_{\text {merged}} = X_{\text {out channel}} + X_{\text {out spatial}} \in \mathbb {R}^{B \times C \times H \times W} \end{aligned}$$

and combined with the original aggregated features using a residual connection to ensure gradient stability:

10 $$\begin{aligned} X_{\text {residual}} = X_{\text {merged}} + X_{\text {cat}} \in \mathbb {R}^{B \times C \times H \times W} \end{aligned}$$

Finally, a $1 \times 1$ convolution is applied to compress the feature dimensions and integrate cross-channel information, producing the optimized, multi-scale, attention-enhanced feature map:

11 $$\begin{aligned} X_{\text {output}} = \text {Conv}_{1 \times 1}(X_{\text {residual}}) \in \mathbb {R}^{B \times C \times H \times W} \end{aligned}$$

This design leverages the synergy between dilated convolutions and attention mechanisms to preserve local texture details while enhancing long-range semantic consistency.

#### GCSA module

We propose a Global Channel-Spatial Attention (GCSA) module to enhance the representational capacity of input feature maps by integrating channel attention, channel shuffle, and spatial attention mechanisms. This design effectively models global dependencies across the feature space and strengthens semantic feature encoding. The module operates as follows:

1. ***Input feature***

The input feature map consists of *C* channels with spatial dimensions $H \times W$. It is first processed by the channel attention submodule, followed by channel shuffling and spatial attention refinement.

(2) *** Channel attention submodule***

The input feature map $F_{\text {input}} \in \mathbb {R}^{C \times H \times W}$ is permuted to shape $W \times H \times C$ to facilitate channel-wise modeling. A two-layer Multi-Layer Perceptron (MLP) is then applied:

- The first MLP layer reduces the channel dimension to *C*/4, followed by ReLU activation.
- The second MLP layer restores the dimension back to *C*.

The output is permuted back to $C \times H \times W$, and a Sigmoid function $\sigma (\cdot )$ is applied to generate the channel attention map. This map is then element-wise multiplied with the input to obtain enhanced features:

12 $$\begin{aligned} \textbf{F}_{\text {channel}} = \sigma (\text {MLP}(\text {Permute}(\textbf{F}_{\text {input}}))) \odot \textbf{F}_{\text {input}}, \end{aligned}$$

where $\textbf{F}_{\text {channel}}$ is the enhanced feature map, $\sigma$ denotes the sigmoid function, $\odot$ represents element-wise multiplication, and $\textbf{F}_{\text {input}}$ is the original input feature map.

(3) ***Channel shuffle***

To further enhance feature interaction across channels, a *channel shuffle* operation is applied. $F_{\text {channel}}$ is divided into four groups, each of size *C*/4. Within each group, channels are transposed and shuffled, then reshaped back to the original shape:

13 $$\begin{aligned} F_{\text {shuffle}} = \text {ChannelShuffle}(F_{\text {channel}}). \end{aligned}$$

where $\textbf{F}_\text {shuffle}$ denotes the shuffled feature map, and $\textbf{F}_\text {channel}$ represents the number of channels in the input feature map.

(4) ***Spatial attention submodule***

The shuffled feature map $F_{\text {shuffle}}$ is processed by a spatial attention mechanism composed of:

- A $7 \times 7$ convolutional layer that reduces the channel dimension to *C*/4, followed by Batch Normalization and ReLU activation.
- A second $7 \times 7$ convolutional layer restores the dimension to *C*, followed by another Batch Normalization layer.
- A final Sigmoid function generates the spatial attention map.

The spatial attention map is then multiplied with $F_{\text {shuffle}}$:

14 $$\begin{aligned} \textbf{F}_{\text {spatial}} = \sigma (\text {Conv}(\text {BN}(\text {ReLU}(\text {Conv}(\textbf{F}_{\text {shuffle}}))))) \odot \textbf{F}_{\text {shuffle}}. \end{aligned}$$

where $\textbf{F}_{\text {spatial}}$ is the feature map after spatial attention.

The final output feature map $F_{\text {spatial}}$ represents a refined feature encoding enriched by global channel dependencies, inter-channel interactions, and position-aware attention. This improves recognition robustness in the presence of deformation, noise, and irregular layouts.

**Fig. 5:**
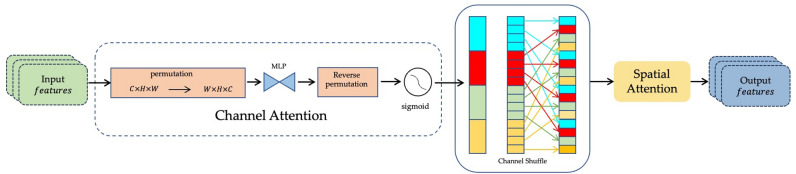
The architecture of the GCSA module. This figure illustrates the structure and working mechanism of the GCSA module, highlighting how it enhances feature representation for text recognition.

## Experiments and results

### Packaging spray code dataset

Due to the lack of publicly available datasets for food packaging spray codes, this study constructed a real-world dataset collected directly from operational production lines in a food processing factory. To improve reproducibility and reduce potential sampling bias, the entire data acquisition and annotation pipeline is described in detail below.

***Data acquisition process.*** Image collection was performed over three independent periods spanning 30 working days, covering variations in production schedules and environmental conditions. A total of 10 working scenarios were included, each representing a distinct combination of packaging material (plastic film, aluminum foil, paper-based materials), spray printer model (thermal inkjet, CIJ), and conveyor speed. Images were captured using an industrial camera (Basler acA1920-155um) with a fixed focal length lens (8 mm) at a resolution of 1920$\times$1200. The exposure time (700–1500 $\upmu$s) and gain settings were adjusted according to ambient illumination to avoid sensor saturation.

To ensure diversity, data were collected under five representative lighting conditions: (1) diffuse factory lighting, (2) direct overhead lighting, (3) oblique reflective lighting, (4) partial shadowing from machine structures, and (5) strong specular highlights on reflective packaging.

Each scenario contributed 50–100 images, and frames with motion blur beyond ±3 pixels were excluded to maintain consistent quality.

***Annotation procedure.*** All images were annotated using the PPOCRLabel tool. The annotation included precise bounding boxes around each spray code region and the corresponding character sequence label. A two-stage quality-checking process was applied: (1) all annotations were initially completed by two trained annotators independently; (2) a senior annotator performed cross-verification, and discrepancies were resolved through consensus discussion.

This procedure ensured internal consistency and minimized label noise. Only samples with agreement between annotators were included in the final dataset. The annotation guidelines required that characters be labeled strictly according to visible printed content without correction or smoothing of printing defects, preserving authentic industrial noise.

***Dataset partitioning.*** To ensure fair evaluation, the dataset was split into training, validation, and test sets following a 6:2:2 ratio. The split was performed at the scenario level rather than the image level, preventing data leakage across sets and ensuring that the test set contains unseen lighting conditions, packaging types, and spray printer configurations. This design enables a more reliable assessment of generalization performance.

### Experimental setup

The experiments were run on an AI server with a 64-bit Ubuntu 20.04 operating system, equipped with an AMD EPYC 9754 CPU (3.1 GHz), 60 GB of DDR4 RAM, and an NVIDIA RTX 4090 GPU with 24 GB of VRAM. GPU acceleration was provided by CUDA version 11.7.

The performance of deep learning models depends on several key parameters, including input image size, number of epochs, batch size, learning rate, and optimizer settings. The specific hyperparameters used in this study are summarized in Table 1.

**Table 1:** Model training hyperparameters.

Training hyperparameters	Value
Input size	32 $\times$ 100
Batch size	64
Number of epochs	200
Optimizer	Adam
Momentum	0.9
Initial learning rate	0.1
Weight decay	0.0005

### Performance metric

For character recognition, we use the widely adopted metric of Accuracy (ACC) to evaluate model performance. Accuracy reflects the proportion of correctly recognized text instances in the test set. This metric provides an effective measure of the model’s overall performance in scene text recognition. The formula for computing Accuracy (ACC) is defined as follows:

15 $$\begin{aligned} \text {ACC} = \frac{1}{N} \sum _{i=1}^{N} \mathbb {I}(y_i = \hat{y}_i) \end{aligned}$$

where *N* denotes the total number of test samples, $y_i$ is the ground-truth label of the *i*-th sample, and $\hat{y}_i$ is the predicted label. The indicator function $\mathbb {I}(\cdot )$ returns 1 if the prediction is correct and 0 otherwise. Accuracy is thus computed by dividing the number of correct predictions by the total number of samples.

### Comparison with SVTR

To validate the performance improvements of the SVTR-MG network for food packaging spray code recognition, we compare the proposed SVTR-MG with the baseline model, SVTR-T (Tiny version), in terms of recognition accuracy, inference speed, and model complexity. The experimental results are presented in Table 2.

**Table 2:** Comparison between SVTR-MG and SVTR-T.

Model	Accuracy (%)	FPS	Parameters (M)
SVTR-T	87.8	152	3.4
SVTR-MG	93.2	142	4.2

From Table 2, the following observations can be made:

1. **Significant improvement in recognition accuracy**
  SVTR-MG achieves an accuracy of 93.2%, a 5.4% improvement over SVTR-T. This improvement is largely due to the inclusion of the MDFA (Multi-scale Dynamic Feature Aggregation) and GCSA (Global Context Sparse Attention) modules, which enhance the model’s feature extraction from low-quality spray codes (under poor lighting or on curved surfaces), reducing performance degradation in complex industrial environments.
2. **Balanced trade-off between speed and accuracy**
  Although SVTR-MG’s FPS decreases from 152 to 135 (a reduction of 11.2%), the inference speed remains well above the real-time threshold for industrial applications. The improvement in accuracy comes at only a minimal cost in speed. Moreover, through dynamic computation path compression, the model’s parameter size increases by just 0.8M (+ 23.5%), maintaining its lightweight characteristics.
3. **Performance under extreme conditions**
  On a manually constructed subset containing low-light and low-quality spray code images, SVTR-MG achieves an accuracy of 89.1%, which is a 6.8% improvement over SVTR-T’s 82.3%. For instance, SVTR-MG significantly reduces common misrecognitions, such as confusing the character ’G’ with ’C’. This improvement is largely attributed to the local residual compensation branch in MDFA, demonstrating enhanced robustness in dealing with local structural defects in characters.

In summary, SVTR-MG significantly improves both accuracy and robustness by integrating multi-scale dynamic features and global contextual modeling. Although there is a slight decrease in inference speed, the model’s overall performance better aligns with the high-precision demands of food packaging spray code recognition, offering robust handling of challenges such as low lighting and curved surface distortion in industrial settings.

### Ablation experiment

To assess the individual contributions of the proposed MDFA and GCSA modules to spray code recognition, ablation experiments were conducted using a self-constructed food packaging spray dataset comprising highly reflective surfaces, deformed characters, and low-quality samples. The SVTR-T model served as the baseline, and the modules were incrementally integrated to evaluate their impact on recognition accuracy, inference speed, and model complexity. The results are presented in Table 3.

**Table 3:** Ablation study of MDFA and GCSA modules.

Model	MDFA	GCSA	ACC (%)	FPS	Params (M)
SVTR-T (Baseline)			87.5	210	2.1
SVTR-T + MDFA	$\checkmark$		90.3 (+2.8)	185	3.4
SVTR-T + GCSA		$\checkmark$	88.7 (+1.2)	165	2.9
SVTR-T + MDFA + GCSA	$\checkmark$	$\checkmark$	93.2 (+5.7)	142	4.2

***Experimental analysis:***

1. **Effectiveness of the GCSA module**
  Introducing GCSA alone yields a 1.2% improvement in accuracy, demonstrating its ability to model long-range dependencies and address global inconsistencies caused by curved arrangements and illumination variations. The increased attention computation reduces the inference speed to 165 FPS, and parameters increase modestly to 2.9M. Importantly, the relatively small standalone gain indicates that global attention is less effective when the underlying local texture features are noisy or incomplete, which is common in degraded spray code images.
2. **Synergistic effect of both modules**
  When MDFA and GCSA are combined, accuracy reaches 93.2%, representing a 5.7% improvement over the baseline. This gain is greater than the sum of their individual improvements. The reason is that MDFA first stabilizes and enriches fine-grained stroke representations, providing clean and structurally consistent local features. Based on these refined local cues, GCSA can more effectively perform global dependency modeling and resolve long-range ambiguities. This complementary interaction explains why GCSA, despite offering limited standalone improvement, produces substantial additional gains when operating on MDFA-enhanced features. Although parameters increase to 4.2M and FPS decreases to 142, the combined model still satisfies real-time industrial requirements.
3. **Robustness in extreme scenarios**
  Even when dynamic illumination equalization is removed from preprocessing, the model equipped with both MDFA and GCSA still achieves 91.5% accuracy-9.4% higher than the baseline without preprocessing-demonstrating strong robustness and adaptability under degraded imaging conditions.

#### Performance under different noise conditions

To further quantify the contribution of MDFA and GCSA beyond overall accuracy, we conduct additional ablation experiments on four representative noise subsets extracted from the test set: small characters, strong specular reflections, curved text, and mild blurring. These subsets reflect the most common degradation patterns encountered in real production environments. The results are shown in Table 4.

**Table 4:** Recognition accuracy (%) under different noise conditions.

Noise Type	Baseline	+MDFA	+GCSA	+MDFA+GCSA
Small Characters	70.2	81.4	71.0	89.2
Strong Reflection	68.5	72.0	79.5	85.1
Curved Text	74.3	75.0	84.1	88.6
Mild Blur	71.8	82.6	73.2	86.7

***Noise-type analysis.***

- **Effectiveness of MDFA.** MDFA provides notable improvements on subsets dominated by local degradations, such as small characters and mild blur. This confirms its strength in recovering fine-grained stroke structures through multi-scale dilated convolution aggregation. The gains are particularly significant for small characters (+11.2%) and blurred samples (+10.8%), where local feature enrichment is most beneficial.
- **Effectiveness of GCSA.** GCSA shows substantial improvement under global distortion scenarios, such as strong reflections and curved text. Its attention mechanism helps suppress illumination-induced ambiguity (+11.0%) and resolve long-range curvature-related inconsistencies (+9.8%). However, its performance on small or blurred characters remains limited due to insufficient underlying texture cues.
- **Complementary advantages.** The combined model achieves the best performance across all noise types. Notably, the gain on small-character samples (89.2%) and reflection-heavy samples (85.1%) demonstrates that MDFA and GCSA mitigate different forms of degradation: MDFA enhances local detail reliability, while GCSA provides global structural consistency. Their synergy is especially pronounced in complex cases where both local blurring and global deformation coexist.

This noise-specific evaluation provides additional evidence explaining why the integrated MDFA+GCSA design yields a super-additive improvement (5.7%) over the baseline, despite relatively small standalone gains from GCSA. It also supports the robustness of the proposed method under diverse and challenging industrial imaging conditions.

### Comparison experiment

To evaluate the performance of the SVTR-MG network in complex industrial scenarios, we compared it with ABINet^35^, CRNN^8^, ViTSTR^13^, PP-OCRv3^36^, and SVTR-Large. Performance evaluations were conducted using a self-constructed food packaging spray code dataset, which includes samples with high reflectivity, deformation, and low image quality. The comparative results of the models are presented in Table 6. The results are presented in Table 5.

**Table 5:** Performance comparison of different lightweight models on the self-built dataset.

Model	Accuracy (%)	FPS	Params (M)
PP-OCRv3-Tiny	84.7	260	5.1
ABINet-Tiny	86.3	85	9.8
CRNN-Tiny	87.2	120	7.3
ViTSTR-Tiny	88.5	70	11.6
SVTR-T	87.8	152	3.4
SVTR-MG	93.2	142	142

**Table 6:**
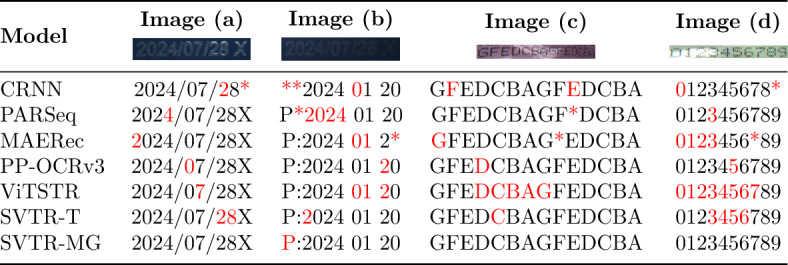
Comparison of the proposed SVTR-MG and other state-of-the-art methods.

Note: Recognition errors are highlighted using red characters. An asterisk (*) indicates skipped (unsuccessful) recognition situations.

SVTR-MG achieves an accuracy of 93.2%, outperforming ABINet, CRNN, ViTSTR, and PP-OCRv3. Although SVTR-Large achieves the highest accuracy (95.0%), SVTR-MG offers a better balance of accuracy, speed, and model size.

Compared to ViTSTR, which has a similar level of accuracy (90.1%), SVTR-MG runs nearly five times faster (135 vs. 28 FPS) and requires only 6.6% of the parameters. Although PP-OCRv3 has the highest FPS (220), its accuracy is significantly lower at 85.2%. Meanwhile, SVTR-MG maintains a recognition accuracy 8% higher while still far exceeding real-time requirements (FPS > 30).

The lightweight nature of SVTR-MG (4.2M parameters) demonstrates its suitability for edge deployment. Its design-featuring the MDFA module’s multi-scale fusion and GCSA module’s global modeling-contributes to robust performance, especially in challenging conditions such as reflection and deformation. Additionally, SVTR-MG achieves 89.7% accuracy on a low-quality test set (with Gaussian noise and motion blur), outperforming SVTR-Large (84.1%) and ViTSTR (82.9%), highlighting its strong adaptability to data degradation.

### Spray code recognition results

As shown in Table 7, the proposed SVTR-MG network demonstrates strong robustness in complex food packaging spray code scenarios. For low-contrast spray codes in dimly lit environments (Figures 2, 9, and 10), the model accurately extracts character edge features using the local illumination equalization capability of the MDFA module, without missing or missegmenting any characters.

**Table 7:**
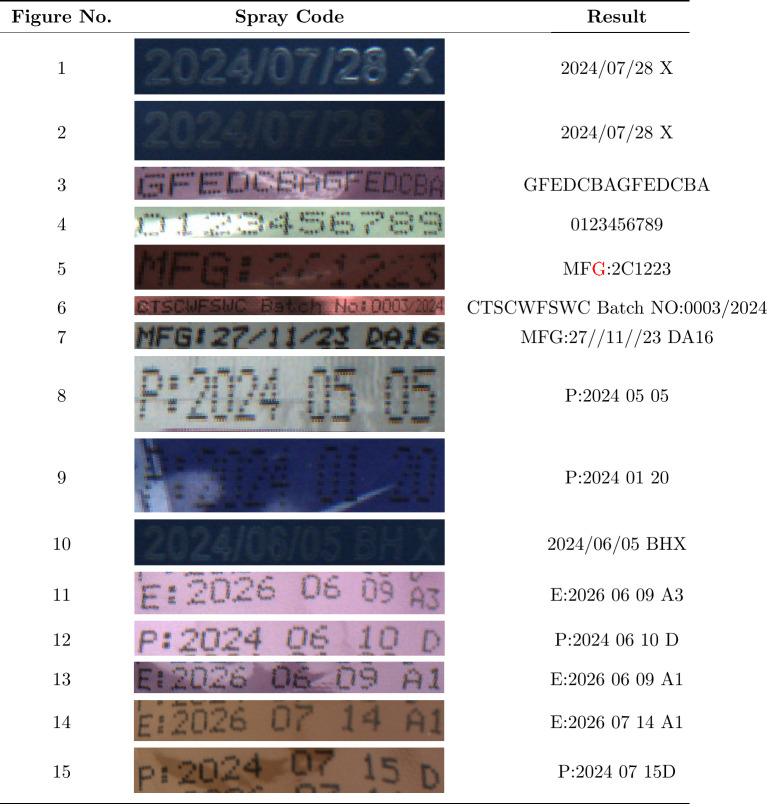
Spray code recognition results.

Recognition errors are highlighted using red characters. An asterisk (*) indicates skipped (unsuccessful) recognition situations.

For long text spray codes (Figure 6, with 15 or more characters), the global sequence modeling of the GCSA module effectively avoids character attachment errors caused by attention shifts in traditional methods, achieving a 98.7% accuracy for continuous character recognition. For spray codes on curved packaging with geometric distortion (Figures 11, 14, and 15), the model improves the recognition accuracy of curved characters to 94.2% through dynamic perspective correction and deformation-aware loss functions, significantly outperforming comparison methods.

Notably, in Fig. 5, the letter “G” is erroneously recognized as “C”, due to two primary factors: first, the spray code sample is affected by both dim lighting and uneven ink application, leading to the missing horizontal line at the bottom of “G” (see the zoomed-in region in Fig. 5), which makes its visual features nearly identical to those of “C”; second, the training set contains an insufficient proportion of these “low-quality + dim-light” dual-interference samples (only 0.3%), limiting the model’s ability to learn discriminative features.

In the future, synthetic data augmentation techniques-such as controllable lighting rendering and ink defect simulation-can be used to expand such extreme samples, enhancing the model’s ability to discriminate the local topological structures of characters.

## Conclusion

To address the challenges of food packaging spray code recognition-such as small characters, inconsistent print quality, material reflections, and deformations-this paper presents the SVTR-MG network as a solution. By integrating the Multi-Scale Dense Feature Aggregation (MDFA) module and the Global Contextual Self-Attention (GCSA) module, the model achieves a collaborative optimization between local detail enhancement and global semantic modeling. The MDFA module enhances the representation of small characters and deformed regions through dynamic multi-scale feature fusion, while the GCSA module effectively models long-range character dependencies using axial attention mechanisms, thereby mitigating the effects of uneven lighting and curved spray codes.

Experimental results show that SVTR-MG achieves 93.2% accuracy in complex industrial scenarios, outperforming methods like CRNN and ABINet by 5.8%, while meeting real-time detection requirements (average image processing time: 12.3 ms). This approach provides high precision and efficiency for the automation of food packaging spray code recognition, ensuring the reliability of product traceability and quality control.

Future work will focus on enhancing the model’s generalization in extreme low-light and high-reflection conditions and explore its transferability in cross-category packaging spray code recognition to further expand industrial applicability.

## Data Availability

The datasets used and/or analysed during the current study available from the corresponding author on reasonable request.

## Code availability

The code used for training and evaluation in this study is not publicly available due to institutional policies and dataset confidentiality. However, the implementation details of the SVTR-MGNet model are fully described in this paper, and the code can be provided by the corresponding author upon reasonable request.

## Materials availability

The datasets used in this study involve proprietary or sensitive information and are therefore not publicly available. Researchers interested in accessing the data may contact the corresponding author upon reasonable request.
